# Preventing Parkinson’s disease in the context of movement disorders: a narrative review of current evidence and future directions

**DOI:** 10.1186/s42466-026-00488-2

**Published:** 2026-04-20

**Authors:** Eva Schaeffer, Jos Steffen Becktepe, Kathrin Brockmann, Carsten Eggers, Eileen Gülke, Elke Kalbe, Tim W. Rattay, Lars Tönges, Tobias Warnecke, Kirsten E. Zeuner, Daniela Berg

**Affiliations:** 1 Department of Neurology, Christian-Albrechts-University of Kiel, UKSH Campus Kiel Arnold-Heller-Straße 3, Haus D, 24105 Kiel, Germany; 2 August-Bier-Klinik, Specialist clinic for neurology and neurorehabilitation, Bad Malente-Gremsmühlen, Germany; 3 Department of Neurodegeneration, Center of Neurology, Hertie Institute for Clinical Brain Research, German Center for Neurodegenerative Diseases, University of Tuebingen, Hoppe Seyler‑Strasse 3, 72076 Tuebingen, Germany; 4 German Center for Neurodegenerative Diseases, University of Tuebingen, Tuebingen, Germany; 5 Knappschaft Kliniken Bottrop, Department of Neurology, Bottrop, Germany; 6 Health and Human Sciences, Macquarie Medical School and Macquarie University Centre for Parkinsons Disease Research, Macquarie University, Sydney, NSW Australia; 7 Department of Neurology, University Medicine Greifswald, Greifswald, Germany; 8 Department of Neurology, University Medical Center Hamburg-Eppendorf, Hamburg, Germany; 9 Medical Psychology | Neuropsychology and Gender Studies & Center for Neuropsychological Diagnostics and Intervention, Faculty of Medicine and University Hospital Cologne, University of Cologne, Cologne, Germany; 10 Center for rare neurological diseases (ZSNE), University Hospital Schleswig Holstein, Campus Kiel, Kiel, Germany; 11 Department of Neurology, St. Josef-Hospital, Ruhr University Bochum, Bochum, Germany; 12 Department of Neurology and Neurorehabilitation, Klinikum Osnabrück - Academic Teaching Hospital of the University of Münster, Münster, Germany

**Keywords:** Prevention, Movement disorders, Parkinson’s disease, Rehabilitation, Risk factors, Early diagnosis

## Abstract

Parkinson’s disease (PD) is the fastest growing neurological disorder worldwide and, together with other movement disorders, belongs to a group of chronic neurological conditions associated with a substantial burden for affected individuals, caregivers, and healthcare systems. Despite significant advances in symptomatic treatment, disease-modifying therapies remain unavailable, shifting increasing attention toward prevention as a key therapeutic strategy. In this narrative we primarily focus on PD, as the epidemiologically most challenging condition and the one for which the most comprehensive evidence base for preventive strategies has been established. Preventive approaches relevant to other movement disorders are briefly discussed to highlight other promising targets requiring further investigations. In recent years major progress has been achieved in the identification of modifiable factors relevant for primary prevention of PD. Well-supported factors include physical activity, adherence to a Mediterranean diet, and caffeine or tea consumption as protective factors, as well as pesticide exposure as a relevant risk factor. Advances in early and prodromal diagnosis of PD have opened new perspectives for secondary prevention. Earlier identification of individuals at risk may enable timely interventions aimed at attenuating early disease progression. However, despite this progress, the systematic implementation of early therapeutic interventions in the prodromal phase remains limited. Evidence is sparse and largely indirect, mainly inferred from later disease onset associated with physical activity and dietary patterns. Similarly, although early diagnosis and treatment of cognitive impairment are clearly recommended by clinical guidelines, they remain insufficiently integrated into routine clinical care. Finally, tertiary prevention strategies are supported by a broad evidence base. Multidisciplinary rehabilitative care models have demonstrated clear benefits in preventing complications, maintaining daily functioning and quality of life. While such rehabilitative approaches are widely implemented, the strong evidence supporting moderate- to high-intensity physical exercise remains insufficiently translated into routine practice. Looking ahead, a key goal for the coming decades is the development of personalized prevention strategies, including beyond other insights into gene-environment interactions and the integration of multi-omics data to tailor interventions to individual risk profiles. Such approaches hold promise to maximize preventive efficacy and reduce the overall burden of PD and related movement disorders.

## Background

Movement disorders are among the most disabling neurological conditions, exerting a profound impact on quality of life and accounting for a considerable share of disability-adjusted life years (DALYs) worldwide [1]. Among them, Parkinson’s disease (PD) represents the most common entity, and in recent years its rapidly rising prevalence has attracted particular attention. PD is now considered the fastest-growing neurological disorder globally. In fact, a recent systematic analysis demonstrated that the number of people living with PD (PwP) almost doubled from 6.2 to 11.8 million within just five years - an increase far exceeding earlier projections [2]. This sharp rise translates into escalating healthcare costs and a substantial societal burden [3]. The challenge is compounded by the absence of disease-modifying or causal pharmacological therapies.

While aging remains the most important risk factor, growing evidence indicates that additional, potentially modifiable, factors also contribute to PD risk. This recognition has shifted attention towards preventive strategies, increasingly emphasized by international health organizations [4, 5]. For example, a 2022 technical brief of the World Health Organization (WHO) on PD explicitly highlighted prevention as a key approach to counter the growing prevalence of the disease [5]. In addition to this central role of primary prevention to reduce the incidence and prevalence of PD, the past years have seen rapidly accumulating evidence for the effectiveness of preventive measures also in secondary and tertiary prevention in PD.

**Fig. 1:**
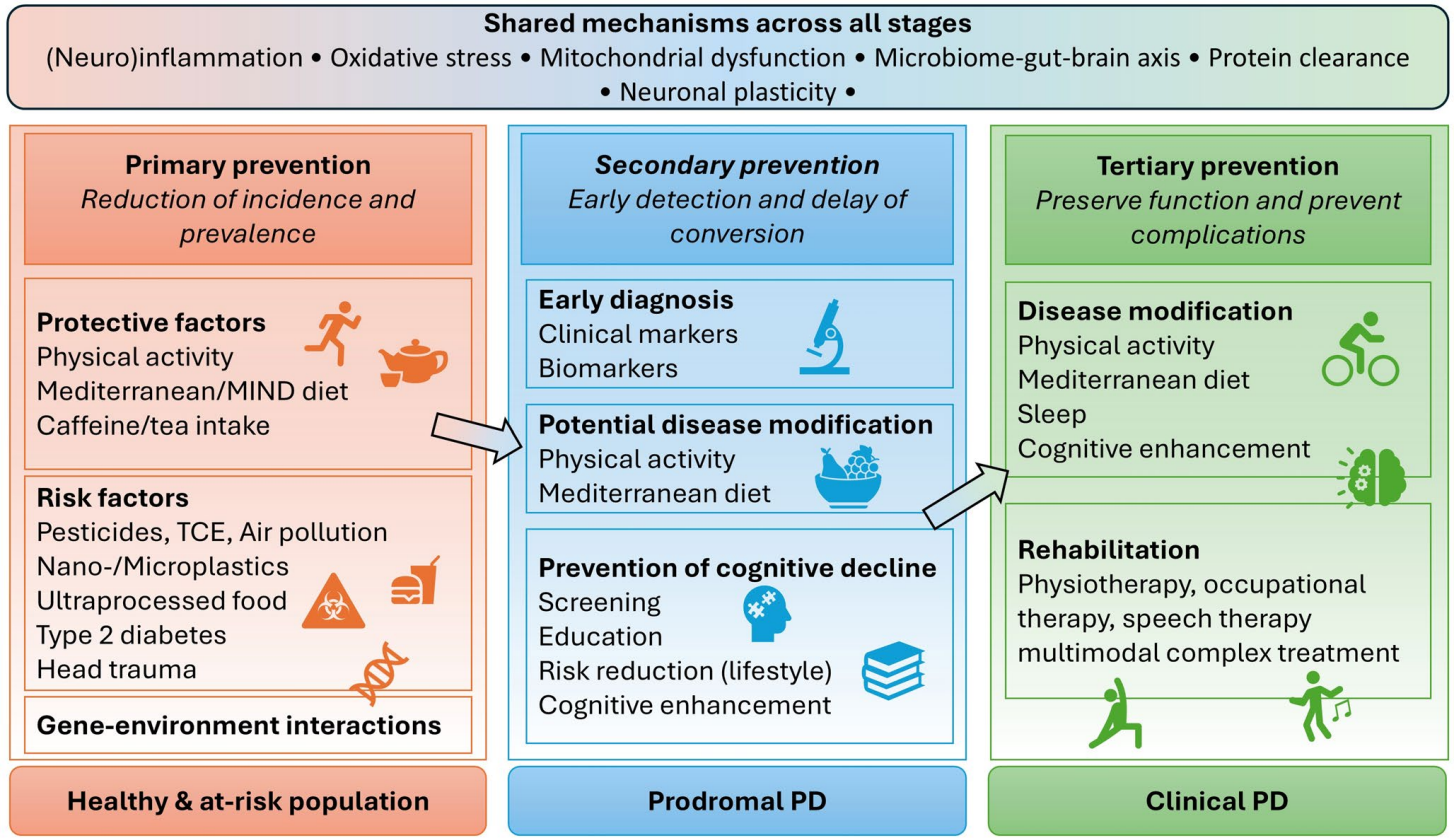
Preventive factors in Parkinson’s Disease. MIND: Mediterranean- DASH - Dietary Approaches to Stop Hypertension Diet Intervention for Neurodegenerative Delay; TCE: trichlorethylene; PD: Parkinson’s Disease

In this narrative review, we summarize current knowledge on primary, secondary, and tertiary prevention, with a particular focus on PD, as the most prevalent and epidemiologically challenging movement disorder. A substantial evidence base for preventive strategies in PD has already been established, highlighting its significant public health relevance (Fig. 1; Table 1). In addition, the review provides a brief overview of preventive approaches in other movement disorders, for which evidence is currently clearly more limited, but which may nonetheless hold considerable potential (Table 2). We acknowledge that pharmacological and surgical treatments can also be considered preventive measures. However, due to their broad scope, these aspects fall outside the focus of the present review, which is intended to concentrate on the identification of modifiable risk factors and non-pharmacological interventions. Beyond summarizing the growing evidence base, we emphasize that effective prevention requires translation into action through coordinated multi-stakeholder engagement, including political support to establish enabling regulatory frameworks and appropriate reimbursement structures.

## Literature Search and Selection

In this narrative review, priority was given to high-quality studies, including systematic reviews and meta-analyses, randomized controlled trials (RCTs), and large prospective cohort studies. Mechanistic studies were additionally included to illustrate pathophysiological plausibility. Smaller RCTs, cross-sectional studies, and other observational studies were also incorporated to provide an outlook on potentially relevant preventive factors; however, these are discussed with lower priority in the text. Tables 1 and 2 further illustrate the gradation of the evidence base. This approach allows for a comprehensive overview of potentially relevant factors while emphasizing those with the strongest current evidence, without strictly adhering to a formal systematic review protocol.

## Parkinson’s Disease

### Primary prevention

In recent years, research on risk and prevention factors for PD has increasingly taken a systematic approach, as exemplified by the work of the Movement Disorder Society (MDS) Task Force on the definition of PD [6, 7]. Since then, further potential factors have been identified, broadening the scope of this field. With regard to modifiable factors, two main domains have emerged: environmental toxins and lifestyle factors. Both interact with endogenous factors, such as comorbidities or alterations of the gut microbiome, which are currently being investigated for their risk-modifying potential [8, 9]. Finally, certain risk factors may be especially relevant for defining high-risk subgroups, i.e. individuals with genetic predispositions, highlighting the importance of stratified or personalized prevention approaches.

#### Modifiable protective factors: physical activity and diet

Robust evidence from epidemiological studies supports an association between higher physical activity and a reduced risk of PD, with risk reductions of up to 60% reported for **moderate to vigorous exercise** [10–14]. Some uncertainties remain, for example, it has been suggested that reduced physical activity in middle age could in some cases be an early prodromal manifestation of subtle motor impairment rather than a causal risk factor. Nevertheless, converging pathophysiological evidence from human and animal studies strongly supports a protective role of exercise for brain health, including reduction of (neuro-) inflammation and oxidative stress, as key pathways in PD pathogenesis, and promotion of neuroplasticity (reviewed in [15]). Some studies have reported sex-specific effects, with a clearer risk reduction observed in men; however, these findings were not confirmed in a recent meta-analysis and therefore remain inconclusive [12, 13, 16].

Diet has emerged as another key modifiable factor. In particular, adherence to the **Mediterranean or MIND diet** (Mediterranean-DASH - Dietary Approaches to Stop Hypertension- Diet Intervention for Neurodegenerative Delay) has been associated with reduced PD risk [17–20]. Evidence from animal and human studies indicates that protective effects are biologically plausible as dietary patterns such as the Mediterranean and MIND diets (including a high amount of polyphenols) also modulate systemic and neuro-inflammation as well as oxidative stress [21–23]. In addition, diet is one of the most influential modulators of the gut microbiome, which has been increasingly recognized as relevant in the context of the gut–brain axis in PD. Diets rich in plant-based foods and fiber can positively influence the composition of the gut microbiome, leading to beneficial metabolic changes, including increased production of short-chain fatty acids (SCFAs), which may in turn contribute to neuroprotection [24, 25].

Beyond the reduction of risk for clinically manifest PD, initial analyses suggest that both lifestyle factors - Mediterranean diet and physical activity - may also lower the risk of prodromal symptoms of PD. In the joint analysis of two longitudinal cohorts, higher physical activity [26] and mediterranean diet [27] was associated with a reduced likelihood of developing prodromal features. However, most of the symptoms assessed (daytime sleepiness, depression, constipation, and body pain) can be influenced by these lifestyle factors independently of the risk for PD. In addition, probable REM sleep behavior disorder (RBD), as a key prodromal marker, was assessed solely via questionnaire; therefore, these findings should be interpreted with caution.

Finally, there is substantial evidence that specific consumables – i.e. **caffeine and tea intake** - have protective effects [28, 29]. The well-established association between smoking and a reduced risk of PD should also be acknowledged [30]; however, smoking cannot be considered a viable prevention strategy due to its well-known harmful effects on multiple other health outcomes. Similarly, another lifestyle factor generally considered harmful - alcohol consumption – has been widely discussed as a potential “protective factor” for PD. Several meta-analyses have reported an inverse association between alcohol intake and PD risk in cross-sectional and longitudinal studies [20, 31–36]. Similar to the association observed with smoking, multiple potential mechanisms and influencing factors have been proposed, including genetic predisposition, premorbid personality or behavioral changes, and direct effects of specific constituents on PD-related pathophysiology [37, 38]. Additionally, the potential “protective effect” of alcohol and tobacco has been debated in the context of possible survivor bias, since studies on PD typically focus on older populations, which may introduce selection effects per se. A recently published meta-analysis on alcohol, smoking, and PD risk further highlighted several influencing factors – such as sex, genetic background, and co-exposures – that were partly inadequately addressed in earlier studies [37]. Taken together, although the epidemiological evidence is diverse, clear biological plausibility remains to be established.

#### Modifiable risk factors: environmental toxins, western diet and head trauma

The strongest evidence for environmental toxins for PD concerns **pesticides** [39]. Multiple epidemiological studies followed by 14 meta-analyses consistently demonstrated a marked increase in PD risk associated with occupational pesticide exposure in general, and with certain agents such as paraquat or maneb in particular [40–42]. Biological plausibility for a causal link mainly derives from animal models, where especially paraquat and rotenone have been used as neurotoxic paradigms inducing selective nigrostriatal degeneration in rodents [43–46]. Additional pesticides (including organochlorines, pyrethroids or chlorpyrifos) have also been implicated in PD-associated neurotoxicity, although the evidence remains less robust. Importantly, findings from animal models (with differing exposure routes and durations) cannot be considered direct proof of human toxicity. However, many aspects remain unresolved, for instance the extent to which non-occupational exposure contributes to risk. Here, association studies have suggested potential links, e.g. with higher PD risk among individuals living in close proximity to golf courses, consuming well water, or residing in pesticide-intensive areas [47–50].

In addition to pesticides, solvents, particularly **trichloroethylene** (TCE), are also being discussed as potential risk factors for PD [6]. In 2023, a cohort study attracted attention, which investigated > 340.000 U.S. veterans stationed at Camp Lejeune, where water supplies had been contaminated with solvents in the 1970–1980 s. Veterans exposed at this site were found to have a ~ 70% increased risk of developing PD decades or prodromal symptoms of PD later compared to veterans of another camp [51]. While these epidemiological findings are striking, again, causality cannot be directly inferred. With respect to biological plausibility, several animal studies have demonstrated PD-like alterations, such as α-synuclein accumulation, following solvent exposure [52, 53]. However, the number and quality of epidemiological and pathophysiological TCE studies remain limited compared to the more extensively investigated pesticide models.

Moreover, increasing industrialization has introduced **air pollution** as an additional emerging environmental risk factor for PD. Especially, fine particulate matter (PM2.5) has been studied in relation to various neurodegenerative diseases, as it may induce neuroinflammation and oxidative stress, either via the lung–brain axis or potentially through direct translocation along the olfactory nerve. However, existing meta-analyses have yielded inconsistent results to date [54–56].

More recently, the potential effects of **nano-/microplastics** have also attracted considerable attention. To date, evidence remains scarce, with only a few initial cell and animal studies investigating their effects on α-synuclein aggregation and mitochondrial function [57–59]. Nevertheless, it should be considered that due to the extensive and near-universal exposure to air pollution and microplastics, even minor individual risk elevations may result in considerable public health implications.

In addition to the increasing burden of environmental toxins, industrialization has also introduced a second emerging risk factor: the so-called Western diet, characterized by a high proportion of **ultra-processed foods** (UPFs). UPFs may be regarded as the counterpart of the Mediterranean or other health-promoting dietary patterns, as they are typically rich in saturated fats, refined sugars, salt, and food additives, while providing only low amounts of fiber, antioxidants, and plant-derived nutrients. Several epidemiological studies have reported a significantly increased risk of PD with high UPF intake, and two recent meta-analyses published in 2025 confirmed this association [20, 60–64]. In line with these findings, two prospective cohort studies also demonstrated links between Western dietary patterns and prodromal symptoms of PD [65, 66]. It should also be considered that **type 2 diabetes mellitus** has long been recognized as an independent risk factor for PD, highlighting the potential role of metabolic dysfunction and systemic inflammation in disease pathogenesis [7].

Finally, **head trauma** has long been discussed as a potentially preventable risk factor for PD. However, meta-analyses and systematic reviews over recent years have so far yielded inconclusive results, requiring the need for further studies [67–72], including more detailed characterization of different types, severities and frequencies of head trauma [73, 74].

#### Importance of risk groups: potential gene-environment interactions

Multiple genetic variants, ranging from rare disease-causing mutations to common low-effect-size single nucleotide variants, as well as environmental factors, have been associated with PD. However, a substantial proportion of disease risk remains unexplained. Consequently, recent research has increasingly focused on gene-environment interactions, which may represent a particularly interesting target for individualized prevention strategies.

As mentioned above, smoking has been inversely associated with PD risk [75]. Interestingly, this association appears to be dependent on the genotype. Carriers of the minor alleles in RXRA-rs420705 (retinoid X receptor A; transcription factor) and SLC17A6-rs1900586 (vesicular glutamate transporter) did not exhibit the protective effect of smoking as compared to frequent allele carriers [76]. Similarly, smoking was shown to modify the previously reported inverse association with the HLA-DRB1-rs660895-G genotype [77]. More recently, a potential interaction between smoking and polygenic risk score (PRS) has been described, with the strongest protective association observed in individuals with a higher PRS [78]. Gene–tobacco interactions appear to be relevant not only for PD risk but also for phenotypic characteristics. PwP with a high genetically defined mitochondrial score showed an earlier age at onset when they were non-smokers [79].

Also, for the previously mentioned inverse association between caffeine consumption and PD risk [80], evidence for gene-environment interactions is accumulating. A recent systematic review including 21 studies with more than 600,000 participants, focusing on PD-related genes and genes involved in caffeine metabolism, reported gene–caffeine interactions for MAPT, SLC2A13, LRRK2, APOE, NOS2A, GRIN2A, CYP1A2, and ADORA2A [81]. Notably, carriers of the Asian LRRK2 p.G2385R risk variant who did not consume caffeine were found to have a 4–8-fold higher risk of PD compared with wild-type caffeine consumers [82]. Similar to tobacco smoking, gene-caffeine interactions appear to be relevant not only for PD risk but also for phenotypical characteristics. In particular, PwP carrying LRRK2 mutations with a high genetically defined mitochondrial score showed an earlier age at onset only if they consumed caffeinated soda [79].

Although this topic partly falls within the field of pharmacological prevention, it should be noted that large epidemiological studies have consistently shown an association between regular use of non-steroidal anti-inflammatory drugs (NSAIDs), particularly aspirin and ibuprofen, and a reduced risk of PD [83]. Regular use of aspirin and ibuprofen has also been associated with reduced penetrance in LRRK2 mutation carriers [84]. This observation is of particular relevance for preventive strategies in PD as inflammation plays a long-standing role in the pathophysiology of both sporadic and genetically associated PD, and most protective lifestyle factors are linked to anti-inflammatory properties. In this context, it is noteworthy that the combination of the three lifestyle factors tobacco smoking, caffeine consumption, and NSAID use appears to have an additive effect on age at onset in sporadic PD. PwP engaging in all three factors showed the latest age at onset compared to those who used none. Moreover, in individuals who did not use any of these three lifestyle factors, a high PRS was associated with a threefold increased risk of PD, an association that was not observed in participants using all three factors [85].

With regard to lifestyle-related factors, physical frailty - defined by weight loss, exhaustion, low physical activity, slow walking speed, and reduced grip strength - has been shown to be associated with PRS in a 12-year longitudinal prospective study. The highest risk of developing PD was observed in individuals presenting both physical frailty and a high PRS, suggesting a relevant interaction between genetic susceptibility and lifestyle-related vulnerability [86].

Finally, the currently most discussed environmental toxin factors are also of high interest for gene-environment interactions, namely pesticides, solvents, and air pollution. The gene PPARGC1α and its encoded protein PGC-1α regulate cellular antioxidant properties and have been associated with PD. An increased PD risk has been observed for the interaction of PPARGC1α-rs68211591 with the pesticides paraquat and maneb [87]. Similarly, PD risk from exposure to paraquat was particularly high in participants lacking GSTT1, a gene encoding glutathione S-transferase T1 which protects against cellular oxidative stress [87]. In the same line of evidence, high exposure to paraquat and maneb resulted in a fourfold increased PD risk in carriers of two or more risk alleles in the dopamine transporter locus in rural California [88]. With regard to solvents, it has been shown that oral exposure to TCE in rats leads to increased LRRK2 activity in the nigrostriatal pathway, accompanied by impaired endolysosomal function [89] In addition to chemical exposures, airborne pollutants have also been implicated in modifying PD risk in the context of genetic susceptibility. A recent case-control study with a meta-analysis design evaluated 1,600 PD participants and 1,778 controls for the interaction of traffic-related air pollution and PRS. Notably, participants with both high PRS and high air pollution exposure showed the greatest risk for PD, with an odds ratio of 3.05, clearly suggesting a synergistic effect [90].

In summary, incorporating gene-environment interactions into future research and clinical practice is crucial to understand the underlying biological mechanisms. This knowledge will help guide personalized prevention strategies, tailored to an individual’s genetic profile and environmental exposures.

### Secondary prevention

#### Disease-modification through lifestyle

In recent years, substantial progress has been made in identifying PD already during the prodromal phase of the disease. The combination of clinical markers and emerging biomarkers is central to early detection strategies. Among clinical markers, isolated RBD (iRBD) is the most predictive prodromal feature. Among biomarkers, the detection of pathological α-synuclein in cerebrospinal fluid and skin using seed amplification assays already allows for high predictive accuracy [91, 92].

Although early detection is considered a central component of prevention, and prodromal cohorts have been established worldwide in recent years, the potential of secondary prevention in PD has so far been largely untapped. Some clinical trials are currently in development or ongoing, but to our knowledge, no RCTs have yet been published [93]. Consequently, no evidence-based conclusions regarding the effectiveness of prevention in the prodromal phase of PD from RCTs can currently be drawn. However, some preliminary observations from epidemiological studies suggest potential lifestyle influences on disease onset. Adherence to a Mediterranean or MIND diet, as well as engagement in moderate (but not vigorous) physical activity, has been associated with a later age at onset in PD, whereas higher consumption of ultra-processed food correlated with an earlier age at onset. These findings might (albeit hypothetically) indicate that a healthy lifestyle could delay disease progression during the prodromal phase, thereby postponing conversion to the clinical stage [19, 94, 95].

The prospect of employing a healthy lifestyle as a disease-modifying strategy in the prodromal phase of PD is closely intertwined with ethical considerations of early detection. Identifying individuals in the prodromal stage by using novel diagnostic approaches entails confronting them with the knowledge of a potentially chronic and burdensome disease, without being able to offer established pharmacological treatment. This dissonance may, at least in part, be mitigated by thorough education about the potential for self-efficacy through lifestyle interventions. In recent years, several survey studies among those affected have shown that such information is perceived as very important in connection with a diagnosis in the prodromal phase [96–98].

#### Prevention of cognitive decline

Mild cognitive impairment and dementia in PwP (PD-MCI and PDD) are frequent and highly debilitating non-motor symptoms [99]. They cause high burden on the individuals affected and their relatives and are related to disease progression and mortality [99]. Also, they constitute a major socioeconomical burden [99]. The estimated point prevalence of PD-MCI is around 40% [100] – with up to one-third of patients already exhibiting PD-MCI at the time of clinical diagnosis [101]. The point prevalence of PDD is 24% to 31% [101] with the risk of dementia increasing with disease duration, from 9% to 27% after 10 years, up to 50% after 15 years, and to 74% after 20 years [102]. With a point prevalence of 36%, subjective cognitive decline, which may be a precursor of manifest cognitive impairment, is also frequent in PwP [103]. Cognitive dysfunction may already occur in the prodromal stage, i.e., in people with iRBD [103]. Notably, up to 90% of people with iRBD develop PD or dementia with Lewy bodies (DLB) within a decade [104].

Despite the frequency and relevance of cognitive impairment in PwP, its prevention has rarely been addressed so far. In a recent concept paper and „call-to-action“ [105], the following terminology in the context of PD was proposed: while interventions to treat cognitive impairment in people with PD-MCI and PDD are assigned to tertiary prevention, all measures taken to prevent the development of cognitive impairment in both clinical PD (with yet no cognitive symptoms) and prodromal PD are regarded as secondary prevention. Given the fact that PwP are in regular neurological treatment, this terminology emphasizes that there is a relevant window for secondary prevention especially in early phases of the disease, in which the majority patients are not (yet) affected by cognitive impairment. For tertiary prevention, recommendations, e.g., in the recently published German PD guidelines [106], include cognitive training and physical interventions to treat PD-MCI, and cognitive stimulation and Acetylcholineesterase inhibitors to treat PDD.

According to the Lancet Standing Commission, 45% of all-cause dementia cases worldwide could be prevented or delayed if all modifiable influencing factors over the lifespan were optimized [107]. The fourteen risk factors indicated include: low education in childhood and adolescence as well as cognitive activity over lifespan, hearing loss, traumatic brain injury, hypertension, high LDL cholesterol levels, excessive alcohol consumption and obesity in middle age, smoking, depression, visual impairment, social isolation, physical inactivity, air pollution, and diabetes in old age. According to a recent update including four further risk factors (poverty, wealth shocks, income inequality, and HIV), even 65% of dementia cases worldwide could be prevented [108]. Although evidence in PwP is less comprehensive than in all-cause dementia, especially Alzheimer’s disease, the concept of ‘cognitive reserve’ also applies. This refers to the brain’s resilience to damage, influenced by lifestyle and medical factors, which affects cognitive functioning [109, 110]. Accordingly, a healthy lifestyle with cognitively stimulating activities, physical activity, and social engagement as well as the management of general health factors (including hearing, vision, and cardiovascular) play an important role in cognitive functioning and the risk of cognitive decline in PwPD.

On the basis of this evidence and the framework proposed by the European Task Force for Brain Health Services ^[111, 112]^ a roadmap for prevention of cognitive impairment in PwP was proposed ^[113] 9^. The “columns” of this strategy include (i) regular assessment of cognitive state, overall risk, and risk factors for cognitive decline, (ii) risk communication and education about modifiable risk factors with standardized procedures, (iii) risk reduction with multi-domain interventions (i.e., cognitive training, physical interventions, diet counselling, management of medical factors) for secondary prevention of cognitive decline, and (iv) cognitive enhancement with cognitive and physical training for tertiary prevention. It is emphasized that this strategy could best be realized in an interdisciplinary care landscape, organized by PD networks, as established or being established in various countries ^[114, 115]^. For risk assessment and risk communication, a standardized protocol was developed ^[116]^. Notably, within the first „PD-Prevention“ trial including multidomain interventions in prodromal and early clinical PD, cognitive decline is addressed, and cognitive training is included ^[117]^.

### Tertiary prevention

#### Tertiary prevention for PD comprises two main components

(i) Rehabilitative interventions aimed at minimizing complications, reducing disability, and improving quality of life. This is primarily achieved through activating therapies such as physiotherapy, occupational therapy, and speech therapy, complemented by optimization of pharmacological treatment and lifestyle interventions. In PD, symptoms associated with disease progression, including balance impairment, falls, and bradykinesia, are targeted for control and alleviation.
(ii) Disease modification: tertiary prevention may also include strategies intended to slow disease progression, potentially relying on neuroprotective mechanisms similar to those pursued in primary or secondary prevention.

#### Preventing complications and preserving Function: Physiotherapy, Occupational therapy, and Speech Therapy

Physiotherapy interventions for PD, based on the European physiotherapy guidelines [118], define five core domains: physical performance and pain, transfers, hand and arm use, balance, and gait. Intervention content includes, for example, range-of-motion and amplitude-based training (e.g., LSVT - Lee Silverman Voice Treatment – BIG, LSVT-BIG), practice of fundamental movement tasks (such as rising from a chair or turning in bed), proprioceptive training (improving balance and preventing falls), general exercise therapy (enhancing cardiopulmonary endurance), and instruction in individualized home-based programs. Relaxation techniques and adjunct physical modalities, including massage and heat applications, may be used as complementary interventions.

A recent review provides detailed guidance on which physiotherapy interventions are effective for specific impairments [119]. In the context of gait disturbances, up to 95 compensatory strategies have been proposed [120], such as stepping initiation with the aid of a walking stick or consciously focusing on increased knee lift amplitude. Specialized physiotherapy for PD has been shown to be associated with fewer complications, reduced mortality, and lower healthcare costs compared with conventional physiotherapy [121].

Occupational therapy interventions, based on established guidelines [122], encompass ten key areas: promotion of self-management, optimization of daily structure and activities, management of stress and time pressure, training of arm and hand motor skills, enhancement of focused attention during everyday tasks, application of cognitive strategies during movement execution, reduction of dual-task demands, use of external cueing strategies, optimization of the physical environment (e.g., counseling on and testing of assistive devices), and education and counseling of family members. A strong emphasis is placed on activity-based therapy targeting activities of daily living, with the development of compensatory strategies for tasks such as eating and personal hygiene. There is partial overlap with physiotherapy approaches, particularly in areas such as fall prevention and range-of-motion training. Evidence suggests that occupational therapy improves quality of life, independence in daily activities, and mobility in people with PD [123, 124].

Speech therapy guidelines primarily focus on the management of speech and swallowing impairments as well as saliva control [125]. Core intervention areas include optimization of food intake with respect to volume and consistency, and amplitude-based speech training, incorporating components of the 14-day LSVT LOUD program, which has been shown to improve effective communication with sustained long-term benefits [126]. Speech therapy targets voice loudness and pitch range, as well as speech intelligibility and speech rate. Following a comprehensive speech and language assessment, individualized therapy should be implemented with the aim of maintaining communication abilities [127]. Management of swallowing disorders focuses on improving both the effectiveness and safety of swallowing. Diagnostic and follow-up assessments may include patient-reported questionnaires, clinical swallowing evaluations, and instrumental methods such as flexible endoscopic evaluation of swallowing (FEES), including FEES performed in the ON/OFF levodopa state. FEES additionally allows for video-assisted swallowing therapy. Adjunctive interventions such as expiratory muscle strength training (EMST) and optimization of dopaminergic medication have also been shown to exert beneficial effects on dysphagia in PD [128].

All three activating therapies described above constitute integral components of PD multimodal complex treatment (PD-MCT), an inpatient, multidisciplinary treatment program implemented in more than 200 hospitals across Germany [129, 130]. PD-MCT combines optimized pharmacological management with activating therapies, as well as structured sports therapy and artistic therapies. Recent evidence indicates that PD-MCT effectively improves motor disability in PD (as assessed by the MDS Unified Parkinson’s Disease Rating Scale, MDS-UPDRS part III) and represents a valuable intervention to prevent or mitigate crisis situations during the disease course [131].

#### Disease-modification through lifestyle

For PD, it has already been demonstrated in several RCTs that physical exercise improves both motor and non-motor symptoms as well as quality of life [132, 133], with a number of systematic reviews and meta-analyses supporting these findings [134–141]. Rehabilitative effects have also been demonstrated, including improvements in muscle strength, functional capacity, cardiorespiratory fitness, balance, dual-tasking and fall risk. These benefits have been achieved through a wide range of training modalities such as endurance training, resistance training or dance interventions [142, 143]. However, it is important to note that most clinical trials primarily investigated short-term benefits over approximately six months. Consequently, these studies cannot provide conclusive evidence regarding long-term preventive or, in particular, disease-modifying effects. However, several studies applying different exercise programs (e.g. MCT and progressive resistance training) extended follow-up to at least 24 months. These studies therefore demonstrated significant and clinically meaningful improvements in motor function, which may already be interpreted as disease modulation [144, 145].

It should also be noted that most of these effective interventions were delivered as supervised training programs, whereas self-management or home-based approaches have been less comprehensive investigated and yielded less consistent results [146, 147]. This results in a marked discrepancy with healthcare reality: while in most countries rehabilitative measures such as physiotherapy and occupational therapy are regularly prescribed, structured and supervised exercise programs are rarely offered. There are, however, studies suggesting that unsupervised approaches can also be beneficial. Retrospective analyses of the longitudinal PPMI and National Parkinson Foundation Quality Improvement Initiative cohorts examined self-reported physical activity levels. They found that moderate to vigorous levels of unsupervised exercise were associated with fewer motor impairments (including postural instability and gait difficulties) as well as improved quality of life after a median follow-up of five and two years, respectively [148, 149]. Finally, it must be acknowledged that the patient’s perspective is crucial, as individuals with PD are the ones who ultimately need to implement these measures. Across many studies, patients have consistently reported a highly positive perception of exercise, which represents an essential prerequisite for therapy acceptance and long-term adherence [150].

In contrast to the already extensive body of evidence on physical activity, clinical nutritional intervention studies in PD have so far been very limited and mainly reported improvements in non-motor symptoms, such as cognition and constipation [151–153]. Particularly noteworthy, however, is that even these small studies have already explored potential pathophysiological mechanisms, including the impact of nutrition on microbiome diversity and enteric inflammation. With regard to long-term observations, a longitudinal study in an elderly population reported that adherence to the MIND diet was associated not only with a lower risk for incident parkinsonism, but also with a slower progression of parkinsonian signs [18]. However, this was not a dedicated observational study in individuals with PD, and results should therefore be interpreted with caution. Moreover, also in the field of tertiary prevention, evidence has only recently begun to accumulate on the potential relevance of UPFs as counterpart of healthy nutrition: Two large prospective cohort studies reported a significant association between UPF consumption and PD mortality [62, 65]. Moreover, the consumption of UPF is closely linked to the established risk factor insulin resistance/diabetes mellitus. Several observational studies, as well as a recent meta-analysis, have already demonstrated a strong association between insulin resistance or diabetes and faster motor progression as well as cognitive decline in PD [154–157].

Finally, long-term effects of individual substances have also been investigated. A meta-analysis on caffeine reported slower PD progression, assessed by the occurrence of dyskinesias and motor fluctuations, the initiation of L-dopa treatment, and progression from disease onset to Hoehn and Yahr stage 3 [29].

In addition to exercise and nutritional components, another lifestyle factor that should be considered in the context of tertiary prevention and neurodegenerative diseases is **sleep**. Investigating this factor with regard to PD risk and disease modification is challenging, as many forms of sleep disturbances are not purely risk factors but rather (prodromal) symptoms of the disease. Nevertheless, healthy sleep appears to be an important contributor to brain health, as suggested by pathophysiological studies on the glymphatic system, which is discussed to play an role in protein homeostasis (including α-synuclein clearance), particularly during deep sleep [158]. A frequently investigated imaging marker of glymphatic function in recent years is the diffusion tensor image analysis along the perivascular space (DTI-ALPS) index. Several prospective and cross-sectional studies have explored the association between the ALPS index and symptom progression in PD, reporting that a lower ALPS index (indicative of impaired glymphatic system function) is significantly associated with faster progression of both motor symptoms and cognitive decline [159–163]. Further clinical studies have reported that low amounts of slow-wave sleep and inadequately treated obstructive sleep apnea are associated with faster motor progression in PD [164, 165].

## Prevention in other movement disorders

Although the evidence for prevention in Parkinson’s disease clearly exceeds that for other movement disorders, substantial potential remains. This review therefore additionally aimes to provide a brief overview of current knowledge and potential preventive targets in other movement disorders in Table 1. The focus here is exclusively on non-pharmacological approaches, although it should be noted that pharmacological and invasive interventions (e.g., deep brain stimulation, botulinum toxin) have a strong evidence base, particularly for tertiary prevention, helping to preserve quality of life and functional independence in advanced stages, as well as to prevent motor and non-motor complications such as contractures, pain and depression [166–172]. In the case of rare genetic diseases with movement disorders, such as leukodystrophies, gene therapies and hematopoietic stem cell transplantation represent a promising avenue for primary and secondary prevention, enabling the earliest interventions in preclinical stages [173–176].

## Conclusion and perspectives

In summary, there is already extensive evidence regarding the impact of lifestyle and environmental factors on *primary prevention* in PD. These findings include modifiable risk factors such as lifestyle and exposure to environmental toxins. Despite the robust evidence, translation into public health and policy strategies for behavioral and structural prevention remains limited. Importantly, preventive efforts should address both individual behavioral interventions (e.g., promoting physical activity) and broader structural prevention measures (e.g., reducing exposure to environmental toxins). Incorporating this knowledge into national and international prevention programs could meaningfully reduce PD incidence and societal burden. Overall, policymakers will play a central role in prevention by shaping regulatory and economic frameworks that reduce exposure to environmental toxins, facilitate healthy lifestyles - for example by making nutritious food more affordable than unhealthy options - and ensure appropriate compensation for early detection and preventive interventions. Additionally, understanding gene-environment interactions is of high interest for individualized primary prevention in PD, providing the basis for precision strategies that can be tailored to a person’s unique genetic and environmental risk profile.

With regard to *secondary prevention*, what is striking is the lack of translation of current knowledge into routine clinical practice and policy strategies. Despite the substantial opportunities offered by secondary prevention, these remain largely underutilized in advancing preventive efforts. The development of novel early-detection markers provides a major opportunity for targeted interventions, provided that early detection is followed by appropriate clinical measures, which is not yet consistently the case. Equally, a clearly underrepresented area in secondary prevention is the implementation of strategies to prevent cognitive deficits. Cognitive decline is one of the most impactful non-motor symptoms in PD, affecting patient quality of life, caregiver burden, and socioeconomic costs. Addressing this gap is essential to ensure that advances in early detection and preventive strategies are translated into meaningful clinical outcomes for patients with PD and other movement disorders.

Moreover, further perspectives emerge for *tertiary prevention*. In addition to the fluid- and tissue-based biomarkers already investigated for the detection of alpha-synucleinopathies, other blood-based biomarkers, such as tau and neurofilament light chain, might in the future be utilized to estimate individual progression patterns, for example the risk of cognitive decline. Furthermore, rapid advances are being made in imaging techniques and digital biomarkers, including at-home solutions such as wearables and smartphone-based assessments, which could further enhance individualized risk stratification and monitoring. The development of these techniques should, however, not be limited to tertiary prevention or empirical prevention, but also enable personalized prevention in all stages of the disease. By applying artificial intelligence and integrating multi-omics data - including genetic, epigenetic, transcriptomic, proteomic, metabolomic, microbiomic, and exposomic information - individual risk profiles could be identified, allowing preventive strategies to be tailored to specific PD subtypes or even at the individual level.

Regarding other movement disorders than PD, it is noteworthy that the evidence for non-pharmacological interventions in most movement disorders lags substantially behind that of PD. Both risk and protective factors have been less well characterized, leaving considerable untapped potential.

Taken together, the potential of preventive strategies in PD and other movement disorders, which are largely chronic conditions associated with high healthcare costs, is considerable, yet many aspects remain insufficiently explored. Future studies as well as funding initiatives should actively support preventive approaches to maximize patient benefit and reduce societal burden.

**Table 1:** Overview of preventive strategies in Parkinson’s Disease

	Primary prevention	Secondary prevention	Tertiary prevention
Parkinson’s Disease	Protective factors $$\begin{aligned} \color{green}{\bullet} \end{aligned}$$Moderate to vigorous exercise [13, 16] $$\begin{aligned} \color{green}{\bullet} \end{aligned}$$Mediterranean or MIND diet [20, 25] $$\begin{aligned} \color{green}{\bullet} \end{aligned}$$Caffeine and tea intake [28, 29] $$\begin{aligned} \color{green}{\bullet} \end{aligned}$$Smoking and alcohol intake ^1^[30, 37] Risk factors $$\begin{aligned} \color{green}{\bullet} \end{aligned}$$Pesticides [177] $$\begin{aligned} \color{yellow}{\bullet} \end{aligned}$$Solvents/trichlorethylene [177] $$\begin{aligned} \color{yellow}{\bullet} \end{aligned}$$Air pollution [177] $$\begin{aligned} \color{yellow}{\bullet} \end{aligned}$$Ultra-processed food [65] $$\begin{aligned} \color{yellow}{\bullet} \end{aligned}$$Traumatic brain injury [67, 68] $$\begin{aligned} \color{yellow}{\bullet} \end{aligned}$$Diabetes mellitus type 2 [7] $$\begin{aligned} \color{red}{\bullet} \end{aligned}$$Micro-/nanoplastics [59]	Protective/positive factors $$\begin{aligned} \color{yellow}{\bullet} \end{aligned}$$Prevention of cognitive impairment: cognitive training/enhancement [110] $$\begin{aligned} \color{red}{\bullet} \end{aligned}$$Moderate to vigorous exercise^2^[94] $$\begin{aligned} \color{red}{\bullet} \end{aligned}$$Mediterranean or MIND diet^2^[95]	Protective/positive factors $$\begin{aligned} \color{green}{\bullet} \end{aligned}$$Physiotherapy, occupational therapy, speech therapy [119, 124, 126] $$\begin{aligned} \color{green}{\bullet} \end{aligned}$$Moderate to vigorous exercise [134, 136–138] $$\begin{aligned} \color{green}{\bullet} \end{aligned}$$Treatment of cognitive impairment: cognitive training [178] $$\begin{aligned} \color{green}{\bullet} \end{aligned}$$[29] $$\begin{aligned} \color{yellow}{\bullet} \end{aligned}$$Mediterranean diet [151, 153] Negative factors $$\begin{aligned} \color{green}{\bullet} \end{aligned}$$Diabetes mellitus type 2 [156] $$\begin{aligned} \color{yellow}{\bullet} \end{aligned}$$Ultraprocessed food [65] $$\begin{aligned} \color{red}{\bullet} \end{aligned}$$Impairment of sleep; obstructive sleep apnoe [163, 164]

Transitions between secondary and tertiary prevention can be gradual. The table presents a selected overview of key original and review publications; additional references are provided in the text

$$\begin{aligned} \color{green}{\bullet} \end{aligned}$$large randomized controlled trials, meta-analyses and systematic reviews

$$\begin{aligned} \color{yellow}{\bullet} \end{aligned}$$prospective cohort studies, cross-sectional studies, small randomized controlled and other clinical trials

$$\begin{aligned} \color{red}{\bullet} \end{aligned}$$Hypertension- Diet Intervention for Neurodegenerative Delay

^1^Both factors cannot represent a viable prevention strategy due to their well-known harmful effects on multiple other health outcome

^2^This association is supported only indirectly through later disease duration

**Table 2:** Overview of preventive strategies in other movement disorders

Tremor*	Primary prevention	Secondary prevention	Tertiary prevention
	$$\begin{aligned} \color{green}{\bullet} \end{aligned}$$ Drug-/toxin-induced tremors: dose reduction or avoidance of tremorgenic drugs and toxins (i.e. lithium, sympathomimetics, amiodarone, valproate, theophylline, ciclosporin) [179–181]	$$\begin{aligned} \color{green}{\bullet} \end{aligned}$$ Enhanced physiologic tremor: treatment of underlying conditions (i.e. hyperthyroidism, hyperparathyroidism, hypocalcemia, hypoglycemia etc.)[182]	$$\begin{aligned} \color{yellow}{\bullet} \end{aligned}$$ Tremor in general: neuromuscular physiotherapy, strength training, limb cooling, Tremor-suppressing orthoses [183] $$\begin{aligned} \color{red}{\bullet} \end{aligned}$$Tremor in general: Avoidance of conditions that exacerbate tremors, such as stress, lack of sleep [184–186] $$\begin{aligned} \color{red}{\bullet} \end{aligned}$$Essential tremor: assistive technology, occupational therapy [187, 188]
Dystonia	$$\begin{aligned} \color{green}{\bullet} \end{aligned}$$ Tardive Dystonia: avoidance of dopamine receptor blocking drugs [189] $$\begin{aligned} \color{green}{\bullet} \end{aligned}$$Cocaine-induced Dystonia: avoidance of Cocaine [190] $$\begin{aligned} \color{green}{\bullet} \end{aligned}$$Task specific dystonia (writer’s cramp, musician’s cramp): avoidance of skilled repetitive movements [191] $$\begin{aligned} \color{yellow}{\bullet} \end{aligned}$$Other medication, drug or neurotoxin induced dystonia: avoidance of respective substances if possible (i.e. manganese, antiepileptic drugs, cholinergic agonists) [192] $$\begin{aligned} \color{red}{\bullet} \end{aligned}$$Task specific dystonia: avoidance of ergonomically challenging equipment in the workplace [193]	$$\begin{aligned} \color{green}{\bullet} \end{aligned}$$Correct diagnosis to detect treatable, symptomatic forms of dystonia or mimics, including [192, 194–196] o Performance of MRI o Screening for immune mediated forms o Screening for infectious, endocrine, metabolic and treatable neurodegenerative diseases o Genetic testing for treatable forms	$$\begin{aligned} \color{green}{\bullet} \end{aligned}$$Cervical dystonia: physiotherapy (/in addition to botulinum toxin) [197, 198] $$\begin{aligned} \color{yellow}{\bullet} \end{aligned}$$ Cervical dystonia: home exercise programs [198, 199] $$\begin{aligned} \color{yellow}{\bullet} \end{aligned}$$ Musician’s dystonia: sensory motor training, task specific motor training [200] $$\begin{aligned} \color{yellow}{\bullet} \end{aligned}$$ Focal dystonia: physiotherapy [201] $$\begin{aligned} \color{yellow}{\bullet} \end{aligned}$$ Musician’s dystonia: behaviour treatment, sensory motor retuning, slow-down exercise; constraint-induced movement therapy, motor control retaining, biofeedback, proprioceptive training [200] $$\begin{aligned} \color{red}{\bullet} \end{aligned}$$ Focal Dystonia: biodfeedback training, voice therapy [201] $$\begin{aligned} \color{red}{\bullet} \end{aligned}$$Dystonia in general: Early detection of suicidal ideations [202]
Huntington’s Disease		$$\begin{aligned} \color{yellow}{\bullet} \end{aligned}$$aerobic exercise, multidisciplinary rehabilitation, general physical activity, computerized cognitive training [203–206]	$$\begin{aligned} \color{green}{\bullet} \end{aligned}$$aerobic exercise, supervised gait training [207, 208] $$\begin{aligned} \color{yellow}{\bullet} \end{aligned}$$balance exercise, physiotherapy, occupational therapy, respiratory training, multidisciplinary rehabilitation (for cognitive function), cognitive training, home-based exercise [207–212] $$\begin{aligned} \color{red}{\bullet} \end{aligned}$$positioning advices and seat adjustments, transfer training, postural control training, caregiver training and education, dental/oral care, speech and swallowing training, nutritional care [208, 210, 213–215]
Ataxia		$$\begin{aligned} \color{green}{\bullet} \end{aligned}$$Spinocerebellar ataxia: neurorehabilitation [216] $$\begin{aligned} \color{yellow}{\bullet} \end{aligned}$$Ataxia with Vitamin E Deficiency: Vitamin E supplementation [217] $$\begin{aligned} \color{red}{\bullet} \end{aligned}$$Episodic ataxia: avoidance of provocative lifestyle factors (alcohol, nicotine, stress) [218]	$$\begin{aligned} \color{green}{\bullet} \end{aligned}$$Genetic Degenerative Ataxia: rehabilitation interventions in general [219]; goal-directed rehabilitation [220], physiotherapy [221] $$\begin{aligned} \color{green}{\bullet} \end{aligned}$$Spinocerebellar ataxia: combination physiotherapy and occupational therapy [222] $$\begin{aligned} \color{yellow}{\bullet} \end{aligned}$$Spinocerebellar ataxia: coordinative physiotherapy, exergames [222] $$\begin{aligned} \color{yellow}{\bullet} \end{aligned}$$Degenerative Ataxia including SCA: coordinative motor training and (biofeedback) speech training for speech production [223–226]
Hereditary Spastic Paraplegia		$$\begin{aligned} \color{red}{\bullet} \end{aligned}$$Early Diagnosis as potential basis for early preventive treatment [227, 228]	$$\begin{aligned} \color{green}{\bullet} \end{aligned}$$Intensive physiotherapy [229] $$\begin{aligned} \color{yellow}{\bullet} \end{aligned}$$Hydrotherapy, robot-assisted gait training, balance training [230, 231]
Leukodystrophies	$$\begin{aligned} \color{green}{\bullet} \end{aligned}$$Newborn screening [232]	$$\begin{aligned} \color{red}{\bullet} \end{aligned}$$avoidance of infections (vaccination) and head trauma [233]	$$\begin{aligned} \color{red}{\bullet} \end{aligned}$$physiotherapy, occupational therapy for spasticity and joint deformities [233, 234] $$\begin{aligned} \color{red}{\bullet} \end{aligned}$$physiotherapy and speech therapy for hypersalivation, dysphagia and malnutrition [233, 234] $$\begin{aligned} \color{red}{\bullet} \end{aligned}$$speech therapy for communication [234] $$\begin{aligned} \color{red}{\bullet} \end{aligned}$$Screening for low bone density, hip dislocation, scoliosis, skin lesions/infections, dental issues, swallowing dysfunction, constipation, urinary dysfunction, respiratory insufficiency, sleep disturbances, pain, seizures, endocrine insufficiency and other systematic affections [234]
Functional Movement Disorders	$$\begin{aligned} \color{yellow}{\bullet} \end{aligned}$$ avoidance and correct treatment of risk factors: physical trauma (sexual abuse), alcohol misuse, chronic pain, psychiatric and psychosomatic disorders (post-traumatic stress disorder, depression, anxiety, suicidal attempts), psychosocial stressors, alexthymia [235–239]	$$\begin{aligned} \color{green}{\bullet} \end{aligned}$$specialized physiotherapy protocol; combined cognitive behavioral therapy with physiotherapy [240] $$\begin{aligned} \color{yellow}{\bullet} \end{aligned}$$Patient centered communication & education [239] $$\begin{aligned} \color{yellow}{\bullet} \end{aligned}$$individualized integrated treatment with (virtual) multidisciplinary interventions/rehabilitation [238, 241–244] $$\begin{aligned} \color{yellow}{\bullet} \end{aligned}$$physical therapy, occupational therapy, speech therapy [239] $$\begin{aligned} \color{yellow}{\bullet} \end{aligned}$$cognitive behavioral therapy, psychological treatment [245] $$\begin{aligned} \color{yellow}{\bullet} \end{aligned}$$body awareness, relaxation practices, expressive therapies, biofeedback [235, 239] $$\begin{aligned} \color{yellow}{\bullet} \end{aligned}$$hypnosis [246, 247] $$\begin{aligned} \color{yellow}{\bullet} \end{aligned}$$promotion of positive prognostic factors (e.g. early and accurate diagnosis strong therapeutic alliance), reduction of negative prognostic factors (e.g. psychosocial stressors [235, 239]) $$\begin{aligned} \color{red}{\bullet} \end{aligned}$$biopsychosocial aetiological physiotherapy [248] $$\begin{aligned} \color{red}{\bullet} \end{aligned}$$tremor retrainment, therapeutic sedation for sever dystonia [249]	$$\begin{aligned} \color{green}{\bullet} \end{aligned}$$ physiotherapy; combined cognitive behavioral therapy with physiotherapy [250, 251] $$\begin{aligned} \color{yellow}{\bullet} \end{aligned}$$Multidisciplinary rehabilitation program [252] $$\begin{aligned} \color{yellow}{\bullet} \end{aligned}$$Tremor: cognitive behavioral therapy [245]

Transitions between secondary and tertiary prevention can be gradual

$$\begin{aligned} \color{green}{\bullet} \end{aligned}$$ large randomized controlled trials, meta-analyses and systematic reviews

$$\begin{aligned} \color{yellow}{\bullet} \end{aligned}$$ prospective cohort studies, cross-sectional studies, small randomized controlled and other clinical trials

$$\begin{aligned} \color{red}{\bullet} \end{aligned}$$ expert opinion, case descriptions, theoretical assumptions

^*^distinct tremor entities independent of other neurological disorders (including neurodegenerative diseases such as Parkinson’s disease)

DBS: deep brain stimulation; MRgFUS: magnetic resonance–guided focused ultrasound; TMS: transcranial magnetic stimulation; SCA: spinocerebellar ataxia

## Data Availability

Not applicable.

## Abbreviations

PD: Parkinson’s Disease
PwP: People with Parkinson’s Disease
WHO: World Health Organization
MDS: Movement Disorder Society
SCFA: Short-chain fatty acids
MIND: Mediterranean Intervention for Neurodegenerative Delay
DASH: Dietary Approaches to Stop Hypertension Diet
iRBD: Isolated rapid eye movement sleep behavior disorder
TCE: Trichloroethylene
UPF: Ultra-processed food
PRS: Polygenic risk score
NSAID: Non-steroidal anti-inflammatory drugs
RCT: Randomized-controlled trail
LSVT: Lee Silverman Voice Treatment
FEES: Flexible endoscopic evaluation of swallowing
EMST: Expiratory muscle strength training
PD-MCT: Parkinson’s disease multimodal complex treatment
DTI-ALPS: Diffusion tensor image analysis along the perivascular space
